# Pentagalloyl glucose from *Schinus terebinthifolia* inhibits growth of carbapenem-resistant *Acinetobacter baumannii*

**DOI:** 10.1038/s41598-020-72331-w

**Published:** 2020-09-18

**Authors:** Micah Dettweiler, Lewis Marquez, Michelle Lin, Anne M. Sweeney-Jones, Bhuwan Khatri Chhetri, Daniel V. Zurawski, Julia Kubanek, Cassandra L. Quave

**Affiliations:** 1 Department of Dermatology, Emory University School of Medicine, Atlanta, GA USA; 2 Molecular and Systems Pharmacology Program, Emory University, Atlanta, GA USA; 3 Center for the Study of Human Health, Emory University, Atlanta, GA USA; 4 School of Chemistry and Biochemistry, Center for Microbial Dynamics and Infection, Institute for Bioengineering and Bioscience, Georgia Institute of Technology, Atlanta, GA USA; 5 Wound Infections Department, Bacterial Diseases Branch, Center for Infectious Disease Research, Walter Reed Army Institute of Research (WRAIR), Silver Spring, Maryland, USA; 6 Emory University Herbarium, Atlanta, GA USA

**Keywords:** Antibiotics, Antimicrobials, Antimicrobial resistance, Small molecules

## Abstract

The rise of antibiotic resistance has necessitated a search for new antimicrobials with potent activity against multidrug-resistant gram-negative pathogens, such as carbapenem-resistant *Acinetobacter baumannii* (CRAB). In this study, a library of botanical extracts generated from plants used to treat infections in traditional medicine was screened for growth inhibition of CRAB. A crude extract of *Schinus terebinthifolia* leaves exhibited 80% inhibition at 256 µg/mL and underwent bioassay-guided fractionation, leading to the isolation of pentagalloyl glucose (PGG), a bioactive gallotannin. PGG inhibited growth of both CRAB and susceptible *A. baumannii* (MIC 64–256 µg/mL), and also exhibited activity against *Pseudomonas aeruginosa* (MIC 16 µg/mL) and *Staphylococcus aureus* (MIC 64 µg/mL). A mammalian cytotoxicity assay with human keratinocytes (HaCaTs) yielded an IC_50_ for PGG of 256 µg/mL. Mechanistic experiments revealed iron chelation as a possible mode of action for PGG’s activity against CRAB. Passaging assays for resistance did not produce any resistant mutants over a period of 21 days. In conclusion, PGG exhibits antimicrobial activity against CRAB, but due to known pharmacological restrictions in delivery, translation as a therapeutic may be limited to topical applications such as wound rinses and dressings.

## Introduction

*Acinetobacter baumannii* is a gram-negative bacterium responsible for a variety of infectious diseases in humans^1^. Carbapenem-resistant *A. baumannii* (CRAB) has been a growing problem in recent decades, categorized as one of five urgent threats by the Centers for Disease Control and Prevention’s 2019 antibiotic resistance threats report, with 700 deaths attributed to it in the US in 2017^2^. CRAB is particularly prominent in healthcare settings among immune-compromised patients, and is difficult to manage due to its high capacity for inherent and acquired resistance, including resistance to desiccation^3,4^. In military medical systems, multidrug-resistant *A. baumannii* contributed to a large number of trauma-related infections in the conflicts in Iraq and Afghanistan^5^. The transfer of multidrug-resistant strains from military personnel to civilian communities presents an additional growing concern^6,7^.

There is currently a lack of new antimicrobials in the drug development pipeline, and many new antimicrobials are specific to gram-positive pathogens^2^. The rise of antibiotic resistance has created a need for new treatments for infections by CRAB and other gram-negative pathogens. One potential source of new antimicrobial drugs is plants used in traditional medicine to treat infections^8^. *Schinus terebinthifolia* Raddi (Anacardiaceae), the Brazilian peppertree, has a variety of uses in traditional medicine, including several dermatological indications^9^. Specifically, the fruit and leaves are used in balms for wounds and ulcers, and laboratory studies have found a variety of antimicrobial activities in *S. terebinthifolia* fruit and leaf extracts^10,11^.

In this study, an extract of *S. terebinthifolia* was subjected to bioassay-guided fractionation for growth inhibition of *A. baumannii*, yielding a bioactive compound, pentagalloyl glucose (PGG), for further investigation as an antimicrobial. PGG has been well studied for antimicrobial activity in gram-positive bacteria, exhibiting growth and biofilm inhibition via iron chelation^12–14^. In light of this previous research and our own isolation of PGG as a bioactive constituent of *S. terebinthifolia* leaves, we subjected PGG to growth inhibitory, anti-biofilm and mechanistic experiments in *A. baumannii*.

## Results

### Bioassay-guided isolation of pentagalloyl glucose

**Figure 1:**
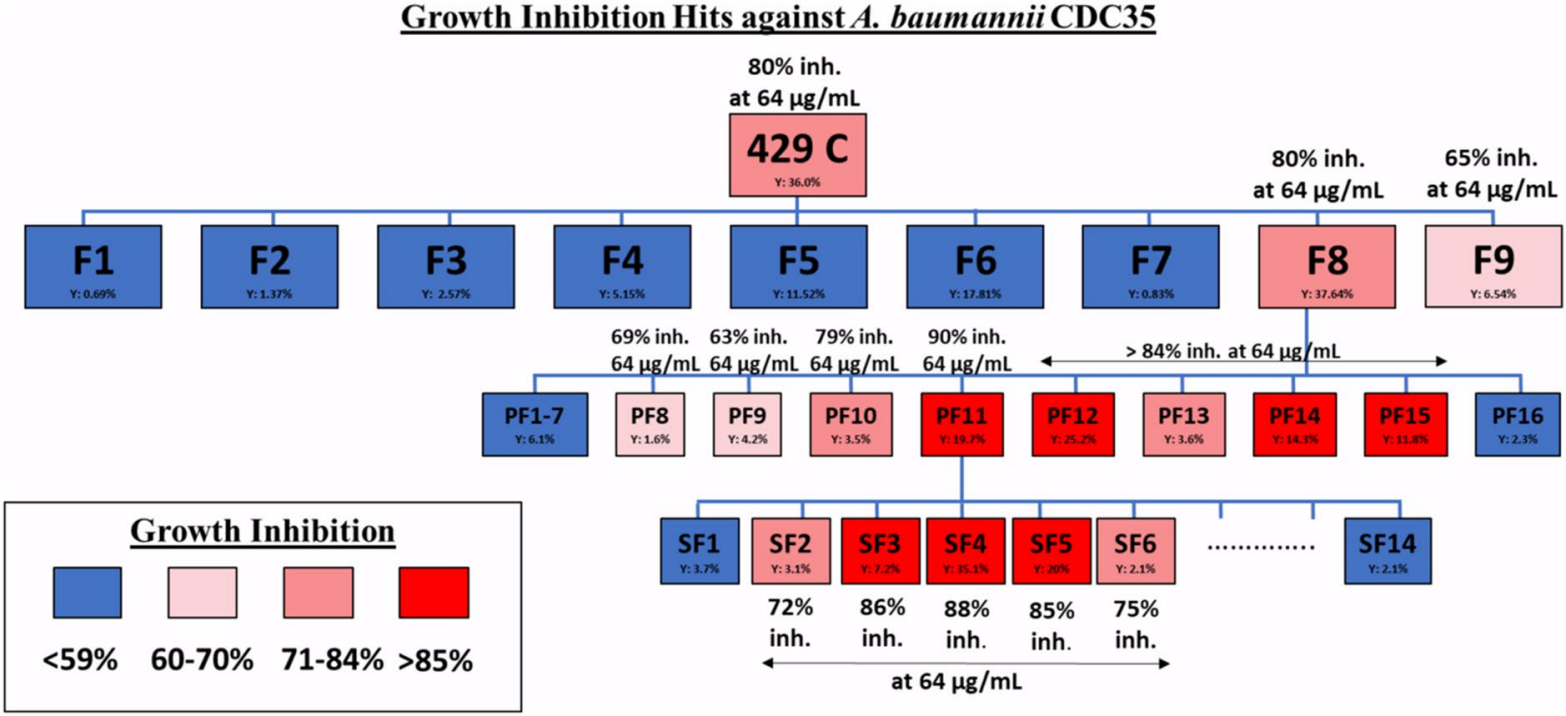
Bioassay-guided fractionation of extract 429 from *Schinus terebinthifolia* leaves using growth inhibition of CRAB.

The Quave Natural Products Library (QNPL) was screened for growth inhibition of CRAB. Of the 1,496 extracts tested, 25 extracts from 19 species exhibited > 80% growth inhibition at a concentration of 256 µg/mL (Supplementary Fig. S1). These 25 extracts were then tested in serial dilution and nine extracts from seven species were chosen for further study based on activity and previous literature. Extract 429, made from the leaves of *Schinus terebinthifolia*, underwent bioassay-guided fractionation for growth inhibition (Fig. 1). Fraction 429C–F8–PF11–SF4 was the most active constituent.

### Chemical characterization

**Figure 2:**
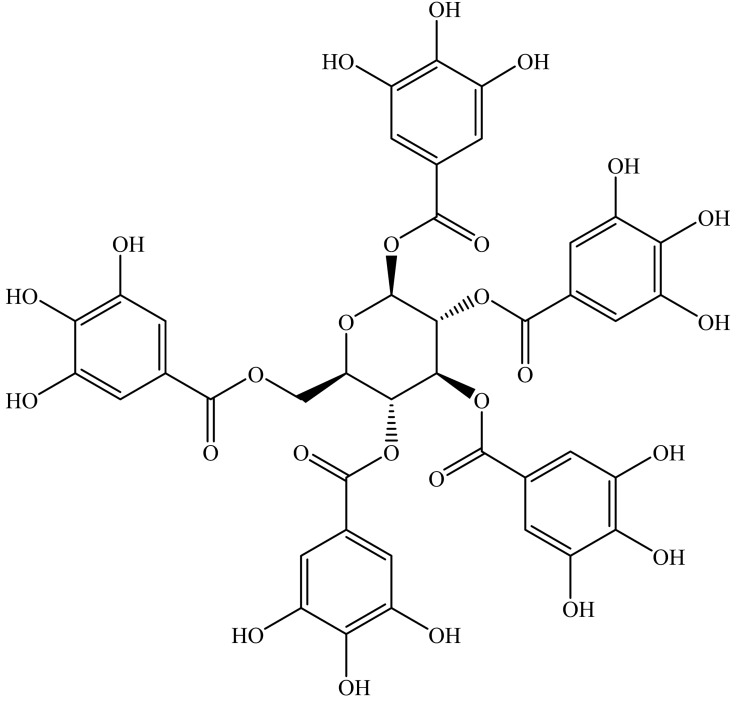
Chemical structure of pentagalloyl glucose (PGG).

Fraction 429C–F8–PF11–SF4 was identified as a pure substance, pentagalloyl glucose (PGG), IUPAC name [(2*R*,3*R*,4*S*,5*R*,6*S*)-3,4,5,6-tetrakis[(3,4,5-trihydroxybenzoyl)oxy]oxan-2-yl]methyl 3,4,5-trihydroxybenzoate (Fig. 2) by nuclear magnetic resonance (NMR) spectroscopy and liquid chromatography–Fourier transform mass spectrometry (LC-FTMS) (Supplementary Fig. S2). The molecular formula for PGG was assigned as C_41_H_32_O_26_ from the (M^+^) ion peak at 939.1096 m*/z*. A standard for PGG obtained from Sigma-Aldrich produced an (M^+^) ion peak at 939.1094 m*/z*. Structure identification was accomplished through comparison of ^1^H and ^13^C NMR spectra to values reported in literature (Supplementary Tables S1, S2; Supplementary Figs. S3–S5) followed by conformation of key connectives using 2D NMR spectroscopic data (Supplementary Figs. S6–S8)^15,16^. The galloyl moieties of PGG produced distinct ^1^H spectral signals with five singlets that integrated to two protons each between δH 6.88–7.14, a product of the symmetry present in the molecule.

### Antibacterial activity of pentagalloyl glucose

**Figure 3:**
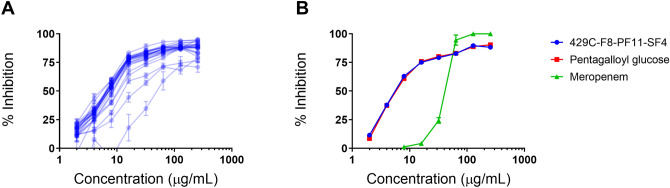
(**A**) Growth inhibition of 24 *A. baumannii* strains by pentagalloyl glucose and (**B**) comparison of *A. baumannii* growth inhibition with 429C–F8–PF11–SF4 and commercially-sourced pentagalloyl glucose. Figure made with GraphPad Prism version 8.3.1 for Windows, www.graphpad.com.

To determine its activity against a wide range of *A. baumannii* strains, PGG was tested for growth inhibition of one susceptible strain of *A. baumannii* and 23 drug-resistant strains (selected for a diversity of resistance profiles, including 19 CRAB strains) (Table 1), yielding minimum inhibitory concentrations (MICs) ranging from 64 to > 256 µg/mL (68 to > 272 µM) and IC_50_s ranging from 8 to 64 µg/mL (8.5–68 µM). PGG produced largely similar dose–response curves against 21 of the 24 *A. baumannii* strains tested (Fig. 3A); much of the variation in MIC values in these 21 strains emerges from small differences in growth inhibition at high concentrations of PGG, since PGG dose–response curves against *A. baumannii* seem to level off around 85–95% inhibition and our definition of MIC is the lowest concentration that exhibits 90% inhibition. PGG was also tested against a panel of other ESKAPE pathogens: *Enterococcus faecium*, *Staphylococcus aureus*, *Klebsiella pneumoniae*, *Pseudomonas aeruginosa*, and *Enterobacter cloacae*. The most potent growth inhibition by PGG was observed in *P*. *aeruginosa*, *A*. *baumannii*, and *S*. *aureus*, with MICs of 16, 64, and 64 µg/mL, respectively (Table 1). The commercially-sourced PGG (Sigma-Aldrich) was found to display identical growth inhibition of *A. baumannii* when compared to PGG isolated from *S. terebinthifolia* (Fig. 3B). Overall, PGG exhibited activity against a wide range of pathogenic bacteria at concentrations ≥ 64 µg/mL.

**Table 1:** Growth inhibition of ESKAPE pathogens by PGG. Abbreviations used are: amikacin (Amk), ampicillin/sulbactam (Sam), cefepime (Fep), ceftazidime (Caz), ciprofloxacin (Cip), erythromycin (Ery), colistin (Cst), gentamicin (Gen), imipenem (Ipm), oxacillin (Oxa), tetracycline (Tet), tigecycline (Tgc), tobramycin (Tob) and vancomycin (Van).

Species	Strain ID	Antibiogram	PGG MIC (µg/mL)	PGG IC_50_ (µg/mL)	Providing source
*Acinetobacter baumannii*	AB5075	Amk^R^,Sam^R^,Fep^R^,Caz^R^,Cip^R^, Gen^R^,Ipm^R^,Mem^R^,Tet^S^,Tob^R^	256	16	Phil Rather
*Acinetobacter baumannii*	ATCC17978	Cst^S^,Mem^S^	> 256	8	Phil Rather
*Acinetobacter baumannii*	Naval-81	Gen^I^	> 256	16	BEI Resources
*Acinetobacter baumannii*	OIFC143	Antibiogram data not available	128	8	BEI Resources
*Acinetobacter baumannii*	NR-9667	Amk^R^,Sam^R^,Fep^R^,Caz^R^,Cip^R^, Gen^R^,Ipm^R^,Mem^R^,Tgc^R^,Tob^R^	256	16	BEI Resources
*Acinetobacter baumannii*	AR Bank #0033	Amk^S^,Sam^R^,Fep^R^,Caz^R^,Cip^R^,Cst^I^,Gen^R^,Ipm^R^,Mem^R^,Tet^S^,Tgc^S^,Tob^R^	> 256	8	CDC AR Bank
*Acinetobacter baumannii*	AR Bank #0035	Amk^S^,Sam^R^,Fep^R^,Caz^R^,Cip^R^,Cst^I^,Gen^R^,Ipm^R^,Mem^R^,Tet^R^, Tgc^S^,Tob^S^	256	8	CDC AR Bank
*Acinetobacter baumannii*	AR Bank #0036	Amk^I^,Sam^I^,Fep^R^,Caz^R^,Cip^R^,Cst^I^,Gen^I^,Ipm^R^,Mem^R^,Tet^R^, Tgc^I^,Tob^R^	> 256	16	CDC AR Bank
*Acinetobacter baumannii*	AR Bank #0037	Amk^S^,Sam^R^,Fep^R^,Caz^R^,Cip^R^,Cst^I^,Gen^R^,Ipm^R^,Mem^R^,Tet^S^, Tgc^S^,Tob^R^	> 256	8	CDC AR Bank
*Acinetobacter baumannii*	AR Bank #0045	Amk^S^,Sam^R^,Fep^R^,Caz^R^,Cip^R^,Cst^I^,Gen^R^,Ipm^R^,Mem^R^,Tet^R^, Tgc^S^,Tob^S^	128	8	CDC AR Bank
*Acinetobacter baumannii*	AR Bank #0070	Amk^S^,Sam^S^,Fep^S^,Caz^I^,Cip^R^,Cst^I^,Gen^R^,Ipm^R^,Mem^R^,Tet^S^, Tgc^S^,Tob^R^	> 256	16	CDC AR Bank
*Acinetobacter baumannii*	AR Bank #0102	Amk^R^,Sam^S^,Fep^R^,Caz^R^,Cip^R^,Cst^I^,Gen^R^,Ipm^S^,Mem^I^,Tet^S^, Tgc^S^,Tob^R^	> 256	8	CDC AR Bank
*Acinetobacter baumannii*	AR Bank #0273	Amk^R^,Sam^R^,Fep^R^,Caz^R^,Cip^R^,Cst^I^,Gen^R^,Ipm^R^,Mem^R^,Tet^R^, Tgc^S^,Tob^R^	128	8	CDC AR Bank
*Acinetobacter baumannii*	AR Bank #0274	Amk^S^,Sam^R^,Fep^R^,Caz^R^,Cip^R^,Cst^I^,Gen^R^,Ipm^R^,Mem^R^,Tet^R^, Tgc^S^,Tob^S^	> 256	64	CDC AR Bank
*Acinetobacter baumannii*	AR Bank #0275	Amk^R^,Sam^R^,Fep^R^,Caz^R^,Cip^R^,Cst^I^,Gen^R^,Ipm^R^,Mem^R^,Tet^R^, Tgc^S^,Tob^R^	> 256	8	CDC AR Bank
*Acinetobacter baumannii*	AR Bank #0277	Amk^S^,Sam^R^,Fep^R^,Caz^R^,Cip^R^,Cst^I^,Gen^R^,Ipm^R^,Mem^R^,Tet^R^, Tgc^S^,Tob^R^	> 256	8	CDC AR Bank
*Acinetobacter baumannii*	AR Bank #0278	Amk^R^,Sam^R^,Fep^R^,Caz^R^,Cip^R^,Cst^I^,Gen^R^,Ipm^R^,Mem^R^,Tet^R^, Tgc^S^,Tob^R^	> 256	8	CDC AR Bank
*Acinetobacter baumannii*	AR Bank #0281	Amk^S^,Sam^R^,Fep^I^,Caz^R^,Cip^R^,Cst^I^,Gen^R^,Ipm^R^,Mem^R^,Tet^R^, Tgc^S^,Tob^S^	128	8	CDC AR Bank
*Acinetobacter baumannii*	AR Bank #0282	Amk^R^,Sam^R^,Fep^R^,Caz^R^,Cip^R^,Cst^I^,Gen^R^,Ipm^R^,Mem^R^,Tet^R^, Tgc^S^,Tob^R^	> 256	16	CDC AR Bank
*Acinetobacter baumannii*	AR Bank #0283	Amk^R^,Sam^R^,Fep^R^,Caz^R^,Cip^R^,Cst^I^,Gen^R^,Ipm^R^,Mem^R^,Tet^R^, Tgc^S^,Tob^R^	> 256	8	CDC AR Bank
*Acinetobacter baumannii*	AR Bank #0284	Amk^R^,Sam^R^,Fep^R^,Caz^R^,Cip^R^,Cst^I^,Gen^R^,Ipm^R^,Mem^R^,Tet^R^, Tgc^S^,Tob^R^	64	8	CDC AR Bank
*Acinetobacter baumannii*	AR Bank #0295	Amk^S^,Sam^R^,Fep^R^,Caz^R^,Cip^R^,Cst^I^,Gen^S^,Ipm^R^,Mem^R^,Tet^R^, Tgc^I^,Tob^S^	> 256	8	CDC AR Bank
*Acinetobacter baumannii*	AR Bank #0299	Amk^R^,Sam^R^,Fep^R^,Caz^R^,Cip^R^,Cst^I^,Gen^R^,Ipm^R^,Mem^R^,Tet^R^, Tgc^S^,Tob^R^	128	8	CDC AR Bank
*Acinetobacter baumannii*	AR Bank #0300	Amk^R^,Sam^S^,Fep^I^,Caz^R^,Cip^R^,Cst^R^,Gen^R^,Ipm^S^,Mem^S^,Tet^R^, Tgc^S^,Tob^R^	256	8	CDC AR Bank
*Enterococcus faecium*	NR-31915	Gen^R^, Van^R^	> 256	128	BEI Resources
*Staphylococcus aureus*	LAC	Ery^S^, Oxa^R^	64	16	Alex Horswill
*Klebsiella pneumoniae*	NR-15410	Amk^S^,Sam^R^,Fep^R^,Caz^R^,Cip^R^, Gen^S^,Ipm^R^,Mem^R^,Tet^S^,Tob^S^	256	8	BEI Resources
*Pseudomonas aeruginosa*	PA01	Antibiogram data not available	16	8	Alex Horswill
*Pseudomonas aeruginosa*	AR Bank #0054	Amk^S^,Fep^R^,Caz^R^,Cip^R^,Gen^R^,Ipm^R^,Mem^R^,Tob^R^	> 64	8	CDC AR Bank
*Pseudomonas aeruginosa*	AR Bank #0064	Amk^S^,Fep^R^,Caz^R^,Cip^R^,Gen^S^,Ipm^R^,Mem^R^,Tob^S^	16	4	CDC AR Bank
*Pseudomonas aeruginosa*	AR Bank #0090	Amk^I^,Fep^R^,Caz^R^,Cip^R^,Gen^R^,Ipm^R^,Mem^R^,Tob^R^	> 64	4	CDC AR Bank
*Pseudomonas aeruginosa*	AR Bank #0092	Amk^R^,Fep^R^,Caz^R^,Cip^R^,Gen^R^,Ipm^R^,Mem^R^,Tob^R^	> 64	4	CDC AR Bank
*Pseudomonas aeruginosa*	AR Bank #0094	Amk^I^,Fep^R^,Caz^R^,Cip^R^,Gen^R^,Ipm^R^,Mem^R^,Tob^S^	64	8	CDC AR Bank
*Pseudomonas aeruginosa*	AR Bank #0095	Amk^S^,Fep^S^,Caz^S^,Cip^R^,Gen^S^,Ipm^R^,Mem^R^,Tob^S^	64	8	CDC AR Bank
*Pseudomonas aeruginosa*	AR Bank #0100	Amk^R^,Fep^R^,Caz^R^,Cip^R^,Gen^R^,Ipm^R^,Mem^R^,Tob^R^	> 64	8	CDC AR Bank
*Pseudomonas aeruginosa*	AR Bank #0103	Amk^I^,Fep^R^,Caz^R^,Cip^R^,Gen^R^,Ipm^R^,Mem^R^,Tob^R^	64	4	CDC AR Bank
*Pseudomonas aeruginosa*	AR Bank #0105	Amk^S^,Fep^R^,Caz^R^,Cip^R^,Gen^R^,Ipm^S^,Mem^R^,Tob^R^	> 64	32	CDC AR Bank
*Pseudomonas aeruginosa*	AR Bank #0108	Amk^R^,Fep^R^,Caz^R^,Cip^R^,Gen^R^,Ipm^R^,Mem^R^,Tob^R^	> 64	8	CDC AR Bank
*Pseudomonas aeruginosa*	AR Bank #0110	Amk^R^,Fep^R^,Caz^R^,Cip^R^,Gen^R^,Ipm^R^,Mem^R^,Tob^R^	64	4	CDC AR Bank
*Pseudomonas aeruginosa*	AR Bank #0111	Amk^R^,Fep^R^,Caz^I^,Cip^R^,Gen^R^,Ipm^R^,Mem^R^,Tob^R^	64	4	CDC AR Bank
*Enterobacter cloacae*	AR Bank #0032	Amk^S^,Sam^R^,Fep^R^,Caz^R^,Cip^S^,Gen^R^,Ipm^R^,Mem^R^,Tet^S^,Tob^I^	> 256	64	CDC AR Bank

Superscript designation: R, resistant; I, intermediate; S, susceptible.

### Biofilm inhibition and eradication

Given PGG’s previously demonstrated anti-biofilm activity against *S. aureus*^13^, PGG was tested for biofilm formation inhibition and biofilm eradication against *A. baumannii* AB5075 at concentration gradients of 0.5–64 (sub-MIC) and 2–256 µg/mL, respectively. PGG did not exhibit any anti-biofilm activity at the concentrations tested, showing mild promotion of biofilm formation relative to vehicle (DMSO) control at 8–64 µg/mL (Supplementary Figs. S9, S10). However, there was no significant difference between PGG and control with regards to biofilm promotion or inhibition at any concentration tested and no dose–response trend was noted. These data support our conclusion that PGG has no effect on biofilm formation or maintenance at this concentration range.

### Cytotoxicity of pentagalloyl glucose

**Figure 4:**
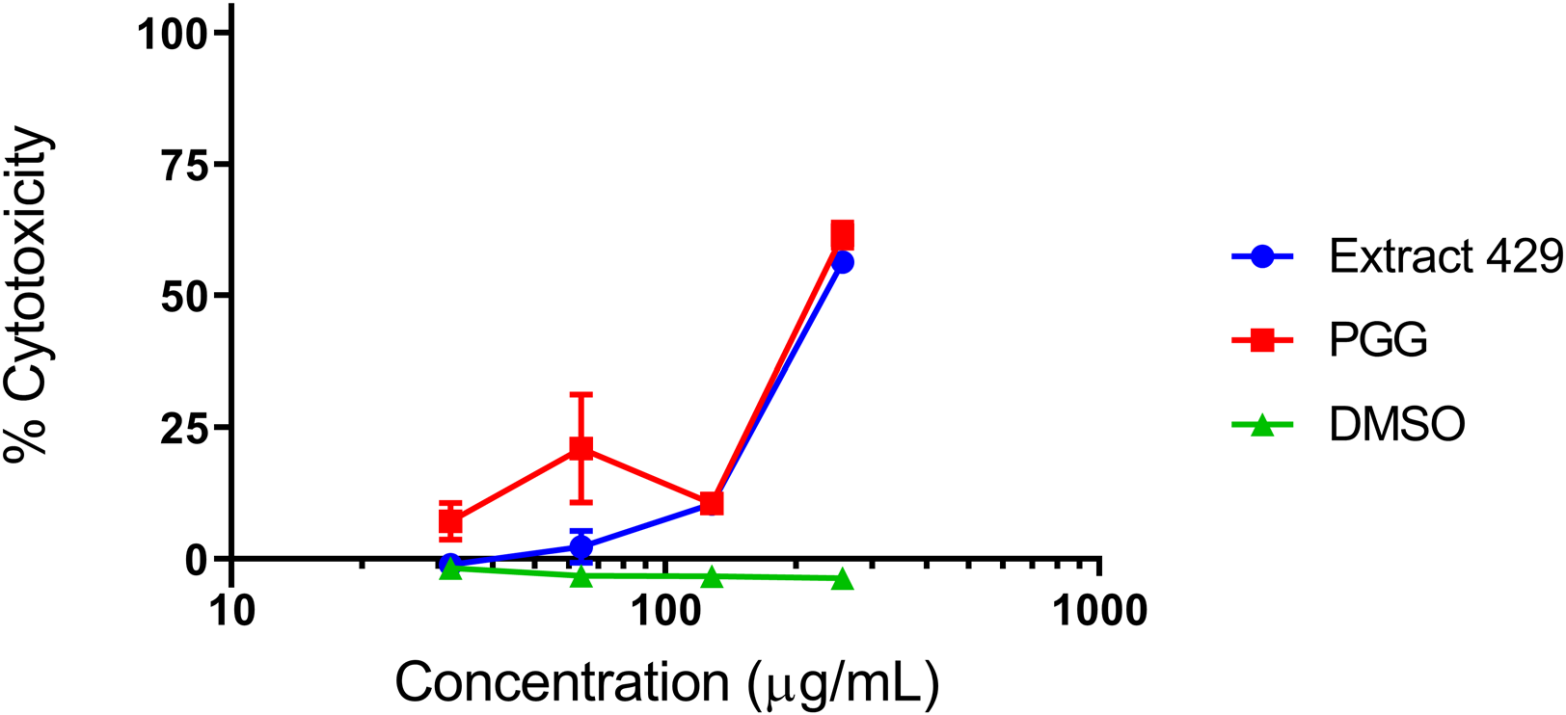
Growth inhibition of *A. baumannii* AR Bank #0035 and human keratinocyte cytotoxicity by PGG and its parent extract 429. Figure made with GraphPad Prism version 8.3.1 for Windows, www.graphpad.com.

Immortalized human keratinocyte cells (HaCaTs) were used in a lactate dehydrogenase (LDH) assay to assess cytotoxicity by PGG and its parents. Both PGG and extract 429 exhibited an IC_50_ of 256 µg/mL (Fig. 4). Using the median IC_50_ (8 µg/mL) for growth inhibition of *A. baumannii* tested in this study, the therapeutic of index of PGG is 32.

### Media supplementation experiments

**Figure 5:**
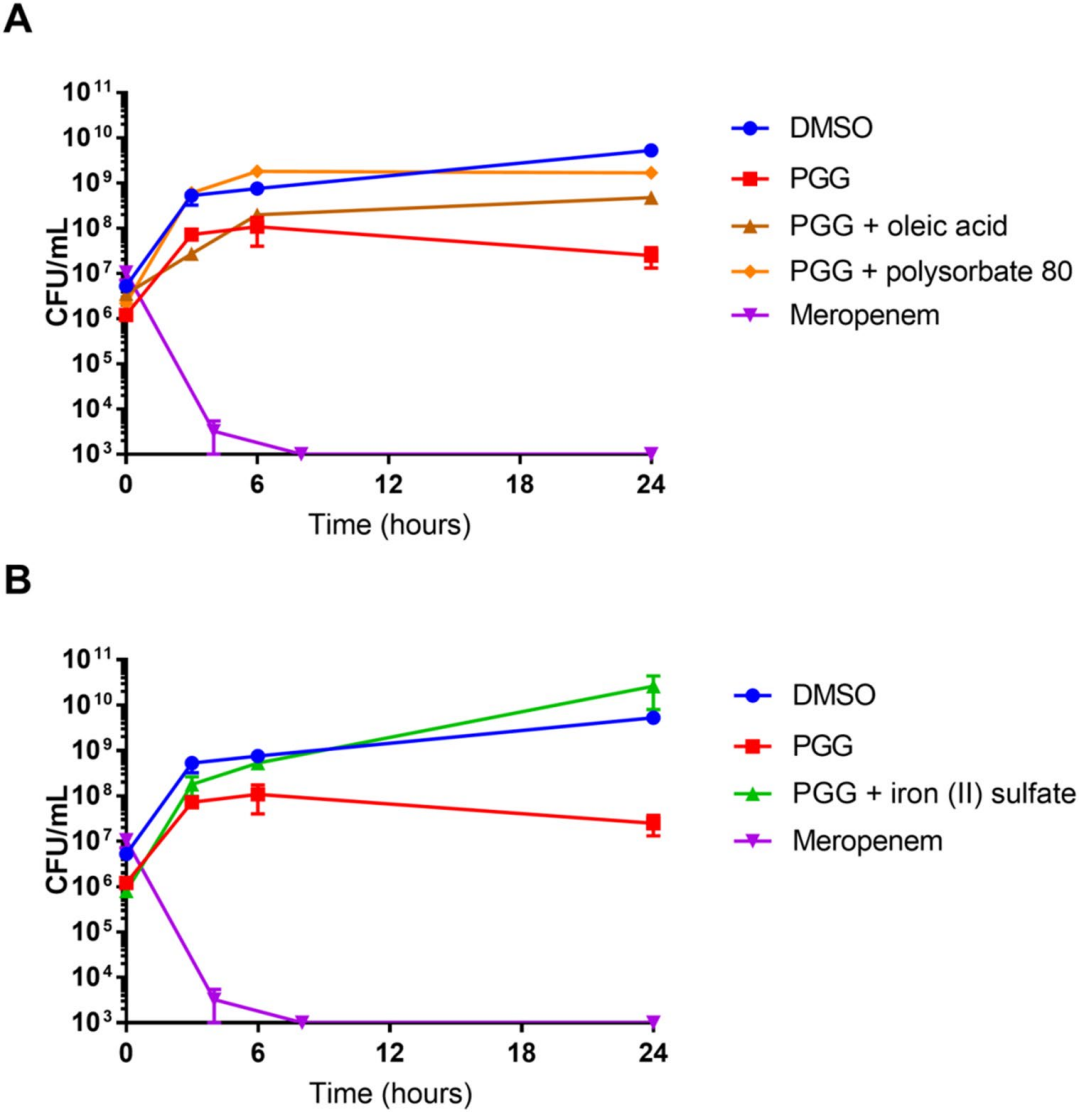
Time-kill assay of *A. baumannii* AB5075 with PGG (256 µg/mL) alone and supplemented with oleic acid, polysorbate 80, and iron (II) sulfate. Meropenem concentration was 64 µg/mL. Figure made with GraphPad Prism version 8.3.1 for Windows, www.graphpad.com.

To elucidate potential mechanisms of action of PGG, *A. baumannii* growth inhibition and time-kill assays were carried out with various supplements in combination with PGG: 0.02% oleic acid, 0.02% polysorbate 80, 1 mM iron (II) sulfate, and 1 mM iron (III) sulfate. In growth inhibition assays of PGG in a gradient of 256–2 µg/mL, oleic acid supplementation produced no change in activity, but polysorbate 80, iron (II) sulfate, and iron (III) sulfate attenuated growth inhibition (Table 2). In time-kill experiments, the CFU/mL curve of PGG alone indicated that PGG’s activity at 256 µg/mL is bacteriostatic rather than bactericidal (Fig. 5). Furthermore, PGG combined with oleic acid, polysorbate 80, and iron (II) sulfate treatments had roughly tenfold, 100-fold, and 1,000-fold higher CFU/mL measurements, respectively, than PGG alone at the 24 h timepoint.

**Table 2:** Effect of fatty acid and iron supplementation on growth inhibition of *A. baumannii* AB5075 by PGG.

Supplement	IC_50_ (µg/mL)	
	PGG	Meropenem
Control	8	32
Oleic acid	8	32
Polysorbate 80	128	32
Iron (II) sulfate	> 256	32
Iron (III) sulfate	> 256	32

### Restoration assays

To test for the restoration of bacterial growth after iron addition, iron (II) and iron (III) sulfate were spread at concentrations of 1 mM on PGG-treated *A. baumannii* AB5075 wells that had no visible colonies after 24 h of incubation. Immediately after addition of iron to the agar, all wells containing PGG obtained a purple colour, increasing in darkness with increasing PGG concentration. After 24 more hours of incubation, colonies were visible in all of the 0.5 × MIC wells, except a single iron (III) supplemented, 0.5 × MIC well; another 24 h of incubation resulted in bacterial growth in all wells and darker media (Supplementary Figs. S11–S12), showing that *A. baumannii* growth inhibition by PGG can be attenuated by addition of iron.

### Resistance studies

To test for spontaneous development of resistance to PGG, *A. baumannii* AB5075 was inoculated on agar plates containing PGG at 0.5 × MIC, 1 × MIC, and 2 × MIC (128, 256, and 512 µg/mL, respectively), and a control plate containing no PGG. After 24 h of incubation, there was a full lawn on the control plate, but no colonies appeared on any of the PGG plates.

**Figure 6:**
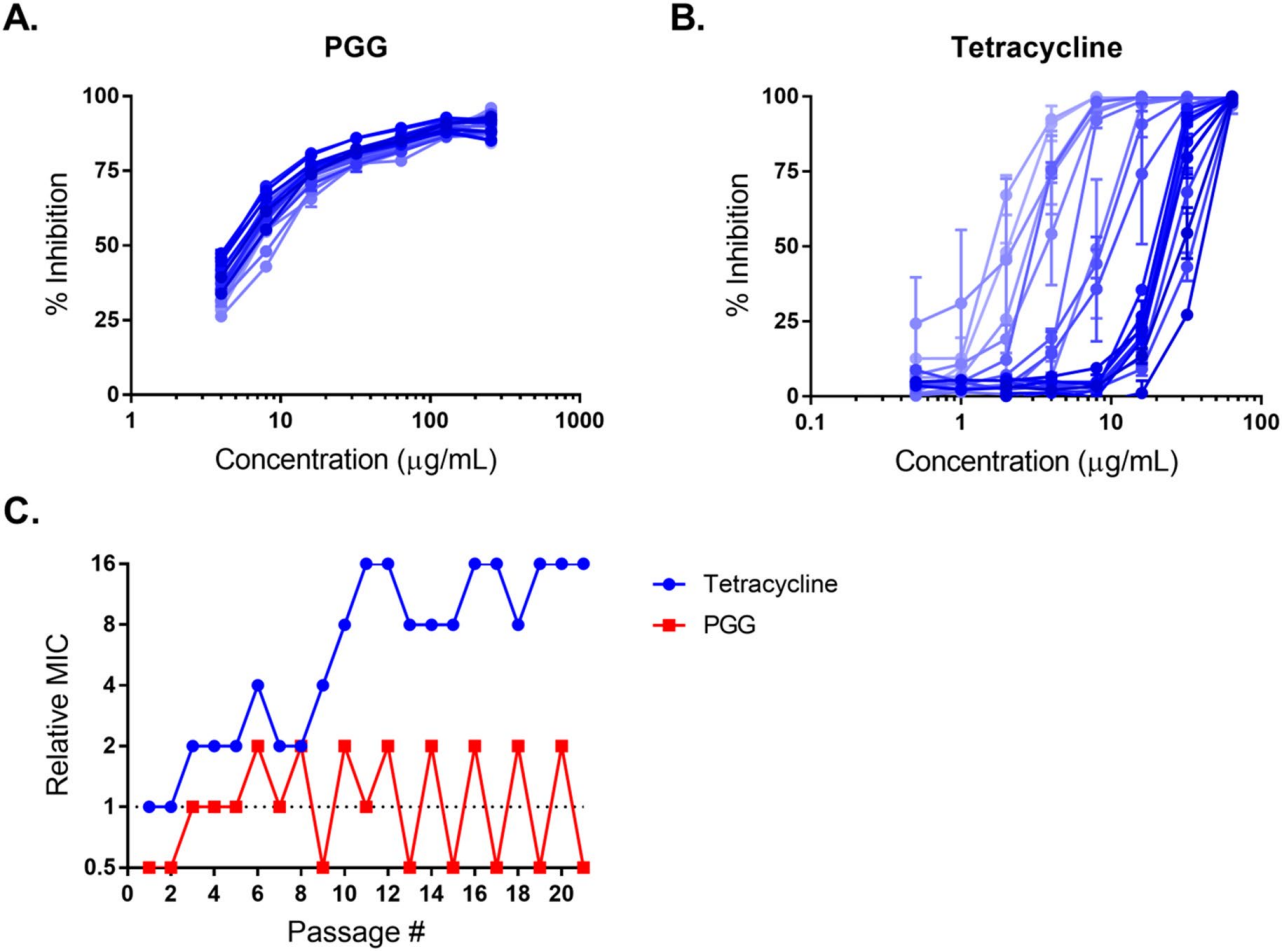
Daily serial passaging of *A. baumannii* AB5075 with PGG and tetracycline, showing change in dose–response curves of (**A**) PGG and (**B**) tetracycline, with darker lines indicating more recent passages, and (**C**) change in MIC of PGG and tetracycline. Base MICs are 256 µg/mL for PGG and 4 µg/mL for tetracycline. Figure made with GraphPad Prism version 8.3.1 for Windows, www.graphpad.com.

Next, evolution of resistance was tested by serial passaging *A. baumannii* AB5075 for 21 days in the presence of a PGG gradient, using culture from the 0.5 × MIC treatment for each subsequent passage. Serial passaging with a tetracycline gradient served as a control. The MIC of PGG remained stable throughout the 21 daily passages, but the MIC of tetracycline increased from 4 µg/mL (susceptible) to 64 µg/mL (resistant) (Fig. 6).

## Discussion

Pentagalloyl glucose (PGG) is a relatively well-known hydrolysable tannin with reported antimicrobial, antiviral, anticancer, antioxidant and antidiabetic properties^17^, but this study is the first to our knowledge to investigate its activity against *A. baumannii*. The bacterial growth inhibition exhibited by PGG in our study is in accordance with PGG’s previously reported broad-spectrum activity, with MICs in the 50–500 µg/mL range and IC_50_ values in the 5–50 µg/mL range^12^.

Previous studies with PGG have attributed its antibacterial activity against *S. aureus* to its chelation of iron, using a colorimetric assay to quantify binding of free iron by PGG and performing bioassays with iron supplementation and 2,2′-dipyridyl, a known iron chelator, as controls^12,14^. Iron chelators have been widely studied as antibacterial agents; in the case of gram-negative bacteria such as *A. baumannii*, potentially therapeutic chelators need to bind iron with higher affinity than the bacterial siderophores but also be distinct enough from these siderophores to not associate with the receptors involved in transporting siderophore-bound iron through the outer membrane^18^. In our experiments with *A. baumannii*, supplementation of the growth media with iron (II) sulfate or iron (III) sulfate completely attenuated growth inhibition by PGG at the concentrations tested, indicating that the previously described chelation mechanism applies in an *Acinetobacter* system.

Restoration of *A. baumannii* growth by addition of iron was also related to the concentration of PGG; the higher the concentration of PGG, the less restoration of bacterial growth was observed after iron supplementation, indicating that PGG interacts with iron in a concentration-dependent manner (Supplementary Fig. S11). This concentration-dependent interaction is also evidenced by optical density readings of PGG in iron (II) and iron (III) sulfate supplemented media (Supplementary Fig. S12). This suggests that PGG's sequestration capabilities, and thus *A. baumannii* inhibition, can be overwhelmed by high iron availability in the environment. Potential applications of PGG as a chelation agent would need to take this into consideration.

We found that 14 *A. baumannii* strains tested had MICs > 256 µg/mL. Speculating on the strain-specific resistance mechanisms based on these MIC values alone is difficult, but the high MIC values seen amongst many of these resistant strains may be attributed to the ability of *A. baumannii* to grow under iron deficient conditions, such as those generated by the iron chelating compound PGG. A previous study by Eijkelkamp et al. found that under iron limiting conditions *A. baumannii* strain ATCC 17978 upregulates genes associated with iron acquisition such as the iron-chelating compound acinetobactin and other related siderophores^19^. The high MIC values (> 256 µg/mL) found for many of the *A. baumannii* strains tested, including the reference strain ATCC 17978 which lacks a resistance profile, may be due to these low iron growth adaptations. It is likely that these same adaptations are found in other PGG resistant *A. baumannii* strains.

Other studies have found that PGG binds to lipopolysaccharide (LPS), a component of the outer membrane of gram-negative bacteria and a causative agent of sepsis^20^. LPS was previously considered essential to gram-negative bacteria, and therefore a target for antibiotics, but some strains, including strains of *A. baumannii*, have been found that can survive without LPS^21^. The attenuation of PGG’s growth inhibition seen in our fatty acid supplementation experiments may be related to PGG’s binding with LPS, but iron supplementation had a larger impact on PGG’s growth inhibition of *A. baumannii*. A previous study has shown that colistin-resistant LPS-deficient *A. baumannii* strains may have higher susceptibility to non-polymyxin drugs due to their decreased membrane integrity^22^; however, this has not been observed in clinical isolates, and compensatory mutations likely occur with respect to colistin-resistance^23–25^. If PGG's mechanism of *A. baumannii* inhibition is indeed through binding of LPS, these considerations may prove useful for the development of drug delivery systems involving PGG. For example, PGG may be utilized prior to an antibiotic, binding to LPS, in order to first disrupt bacterial membrane integrity.

PGG’s lack of anti-biofilm activity against *A. baumannii* contrasts with its documented anti-biofilm activity against *S. aureus*^13,14^. However, this discrepancy can be attributed to differences in biofilm mechanisms between *A. baumannii* and *S. aureus*; removal of iron prevents biofilm formation in *S. aureus*^14^, but studies with *A. baumannii* have shown an indifferent response^26^ or even increased biofilm formation when iron is limited^27^. Furthermore, previous experiments with deferasirox, a clinical chelator with high affinity to iron, found no significant anti-biofilm activity on *A. baumannii*, suggesting that *A. baumannii* is highly capable of sequestering iron in biofilm^28^. However, every iron chelator has independent activity, and some results have been strain dependent^29,30^. Also, limiting iron negatively impacts motility^31^, which has been determined to play a key role in virulence^32,33^. From these results, it appears that PGG is capable of chelating iron from the medium to inhibit planktonic growth, but a physical or mechanistic difference in the bacteria's iron withholding capacity interferes with PGG's iron chelation capacity in biofilm. These results also reinforce the concept that differential responses between bacterial species can prevent antibacterial activity in one species from being generalized to other species without specific testing.

Through time-kill assays with PGG, we found it to be bacteriostatic against *A. baumannii*. Testing PGG against a panel of 24 *A. baumannii* strains with a wide range of resistance profiles showed that PGG’s activity is generally consistent between strains, and similar growth inhibition activity in a panel of ESKAPE pathogens demonstrated that PGG has activity against both gram-positive and gram-negative bacteria. Passaging *A. baumannii* 21 times in the presence of PGG did not stimulate any spontaneous resistance, in contrast to the same passaging with tetracycline—another bacteriostatic agent—which produced a resistant phenotype (16-fold increase in MIC) after 10 passages. Together, these experiments indicate that PGG has broad-spectrum antibacterial activity, likely inhibiting growth by chelating iron, and that resistance to this activity does not develop quickly in *A. baumannii*.

In cytotoxicity assays with human keratinocytes, PGG exhibited an IC_50_ of 256 µg/mL, yielding a therapeutic index of 32 for PGG’s growth inhibition of *A. baumannii*. PGG may therefore be useful as a topical therapy for infections, and the anti-infective traditional use of *S. terebinthifolia* and other members of the Anacardiaceae containing PGG is supported^10,12^.

Further development of PGG as a potential drug requires more mechanistic and pharmacokinetic/pharmacodynamic study. Previous pharmacological studies of PGG, recently reviewed by Patnaik et al.^34^, have found low oral bioavailability^35^ but potential for intravenous administration^20,36^ with possibilities for nanoparticle-based^37^ and microbubble-based delivery^34^.

PGG’s lack of specificity, typical to tannins^38^, may limit or at least modulate its use as an antimicrobial, but its activity against CRAB, an urgent healthcare threat^2^, supports its further investigation for anti-infective therapy.

## Methods

### Plant extracts

The leaves of *Schinus terebinthifolia* Raddi (Anacardiaceae), which were selected from a primary screen of the Quave Natural Product Library (QNPL), were collected from private property in DeSoto County, Florida, in November 2017. Plant samples were identified and catalogued by Dr. Cassandra Quave at the Emory Herbarium (GEO, Atlanta, Georgia) where voucher specimens were deposited for reference (CQ-651) and are accessible for view online via the SERNEC portal^39^. Fresh plant samples were dried in a dehumidified cabinet. Dried plant material was ground using a Wiley Mill Plant Grinder and filtered through a 0.5 mm mesh sieve. Powdered plant samples were subjected to two rounds of maceration in 80% aqueous ethanol for 72 h, filtered, and then dried via a rotary evaporator. Crude plant extracts were partitioned using a successive liquid–liquid partitioning scheme. Extraction solvents used were: hexanes, ethyl acetate, n-butanol, and water and were labelled B, C, D, and E according to solvent, respectively. Dried crude plant extracts were stored in – 20 °C until further use.

### Isolation of bioactive compounds

*Schinus terebinthifolia* extract 429 underwent bioassay-guided fractionation as previously described^40^. The ethyl acetate partition 429C (13.56 g) was fractionated using a 330 g silica column (RediSep, Teledyne ISCO) via normal phase flash chromatography (Combi Flash Rf + Lumen, Teledyne ISCO) utilizing the following hexane:ethyl acetate gradient: 3 column volumes (CV) 100:0, 30 CV gradient to 0:100, and 32 CV isocratic 0:100. The bioactive fraction 429C–F8 (5.10 g, 37.6% yield), was eluted between 27.5 and 40.0 CV’s. All subsequent preparative high-performance liquid chromatography (Prep-HPLC) were carried out using an Agilent Technologies 1260 Infinity II LC System (CA, USA) equipped with an Agilent Technologies 1200 Infinity Series Diode Array Detector detecting at 214 nm and 254 nm. The column used for all subsequent Prep-HPLC purifications was an Eclipse XDB-C18 5 μm pore, 30 × 250 mm reverse phase column (Agilent). Fraction 429C–F8 was fractionated further via Prep-HPLC using a mobile phase of 0.1% (vol/vol) formic acid in water (A) and 0.1% (vol/vol) formic acid in acetonitrile (B) at a flow rate of 42.5 mL/min. To fractionate 429C–F8, the following gradient (A:B) was used: 0 min (98:2), 3 min (98:2), 11 min (90:10), 38 min (81:19), 58 min (81:19), 58.1 min (80:20), 68 min (80:20), 75.5 min (21:79). The bioactive fraction 429C–F8–PF11 (137.8 mg, 19.7% yield), was eluted between 36.75 and 41.17 min. Fraction 429C–F8–PF11 was fractionated further via Prep-HPLC using a mobile phase of 0.1% (vol/vol) formic acid in water (A) and 0.1% (vol/vol) formic acid in methanol (C) at a flow rate of 42.5 mL/min. To fractionate 429C–F8–PF11, the following gradient (A:C) was used: 0 min (85:15), 10 min (65:35), 25 min (65:35), 25.1 min (60:40), 35 min (60:40), 35.1 min (2:98), 47 min (2:98). The bioactive fraction 429C–F8–PF11–SF4 (53.6 mg, 36.0% yield), was eluted between 13.0 and 14.0 min.

### NMR spectroscopy

A 14.1 T (600 MHz for ^1^H, 150 MHz for ^13^C) Bruker Ascend III HD NMR spectrometer with a 5 mm prodigy cryoprobe or an 18.8 T (800 MHz for ^1^H, 200 MHz for ^13^C) Bruker Avance III HD NMR spectrometer with a 3 mm triple resonance broadband cryoprobe were used to acquire NMR spectra (^1^H, ^13^C, COSY, HSQC, and HMBC). PGG was dissolved in either DMSO-d_6_ or CD_3_OD for NMR spectral acquisitions and referenced to solvent residual peaks (δ_H_ 2.50 or 3.31 for DMSO-d6; δ_C_ 39.52 or 49.00 for CD_3_OD). MestReNova 12.0.0 was used to process and analyze spectra.

### LC-FTMS analysis

A sample of 429C–F8–PF11–SF4 and a standard of pentagalloyl glucose (Sigma-Aldrich, St. Louis, MO) was analysed by liquid chromatography–Fourier transform mass spectrometry (LC-FTMS) using a Thermo Electron LTQ-FT Ultra MS (Thermo Scientific) coupled to a Shimadzu LC (Columbia, MD) equipped with a Shimadzu autosampler and Dionex (San Jose, CA, USA) HPLC pump and diode-array detector. The stationary phase was an Eclipse XDB-C18 5 μm pore, 30 × 250 mm reverse phase column (Agilent). A 10 μL injection of sample was applied for each run. Mobile phases consisted of 0.1% (vol/vol) formic acid in water (A) and 0.1% (vol/vol) formic acid in acetonitrile (B) at a flow rate of 1.0 mL/min. The following gradient (A:B) was used: 0 min (70:30), 7 min (65:35), 13 min (65:35), 13.01 min (60:40), 20 min (60:40), 20.01 min (0:100), 30 min (0:100), with a return to starting conditions at 30.01 min (70:30) until 40 min (70:30). Mass spectrometry samples were ionized by positive electrospray ionization at the following conditions: 5 kV voltage applied to needle, capillary voltage of 45 V, capillary temperature of 200.0 °C, and tube lens voltage of 100 V. Mass spectrometry data was processed with Freestyle 1.6 software (Thermo Scientific). The predicted formula and mass were taken for 429C–F8–PF11–SF4 and the PGG standard according to the peak compound signature as determined previously by analytical HPLC of 429C–F8–PF11–SF4 compared to a standard of PGG.

### Bacterial growth conditions

Bacteria were maintained on tryptic soy agar (TSA) plates and grown in cation-adjusted Mueller Hinton broth (CAMHB) for experiments according to CLSI guidelines^41^. Experimental cultures were incubated at 37 °C in a humidified chamber. AR Bank strains were obtained from the FDA-CDC Antimicrobial Resistance Isolate Bank^42^; a full list of the strains tested is available in Table 1. *A. baumannii* AB5075, the primary strain used in the following assays, is a model strain that is more virulent and is a representative ST2 strain. ST2 is the dominant MLST-type clone worldwide, responsible for the majority of outbreaks in Europe, Middle East, South America, and Asia, and these strains are often the most antibiotic resistant as well^43–48^.

### Growth inhibition

Bacterial growth inhibition was determined by microbroth dilution according to CLSI guidelines^41^. Bacteria were grown overnight in TSB and standardized to 5 × 10^5^ CFU/mL in CAMHB to make the experimental culture. Treatments were added in triplicate and absorbance of experimental wells was measured at 600 nm with a BioTek Cytation3 plate reader before and after incubation (22 h for *A. baumannii* and 18 h for other ESKAPE species). Percent growth inhibition was calculated relative to vehicle control (DMSO); the minimum inhibitory concentration (MIC) was determined as the lowest treatment concentration with > 90% inhibition and the IC_50_ was the lowest concentration with > 50% inhibition. Data was analysed using Microsoft Excel and figures were created with GraphPad Prism version 8.3.1. Positive controls were meropenem and colistin for the 24-strain *A. baumannii* panel and meropenem and gentamicin for other growth inhibition experiments. A media blank was included in each experiment to test for contamination and each experiment was performed twice on separate days.

For supplementation experiments, oleic acid and polysorbate 80 were added to respective experimental cultures to a concentration of 0.02% vol/vol^49^, and iron (II) sulfate and iron (III) sulfate were dissolved in deionized water and added to a concentration of 1 mM^12^.

### Time-kill

Time-kill methods were adapted from NCCLS guidelines^50^. PGG and meropenem were added at MIC concentrations (256 and 64 µg/mL, respectively) to *A. baumannii* AB5075 at 1 × 10^6^ CFU/mL in CAMHB [alone or supplemented with 0.02% oleic acid or polysorbate 80 or 1 mM iron (II) sulphate]. At 0, 3, 6, and 24 h timepoints, 10 µL culture was removed from each of four replicates, serially diluted tenfold in sterile PBS, dispensed on TSA, and incubated to determine CFU/mL.

### Biofilm inhibition and eradication

Methods for *A. baumannii* biofilm inhibition and eradication were adapted from Tipton et al.^51^. Briefly, *A. baumannii* AB5075 cells were grown in Luria–Bertani broth (LB) for 24 h in the presence of treatment in the case of biofilm inhibition and for 24 h pre-treatment and 24 h post-treatment in the case of biofilm eradication. After incubation, biofilms were fixed with ethanol, stained with crystal violet, gently rinsed with water, and eluted with 33% acetic acid. The absorbance of each well was measured at 595 nm.

### Mammalian cytotoxicity

Toxicity to immortalized human keratinocytes (HaCaTs) was assessed using an LDH cytotoxicity assay kit (G-Biosciences, St. Louis, MO, USA) as described previously^52^. Briefly, cells were standardized to 4 × 10^4^ and incubated in 96-well plates for 48 h to allow for seeding, after which media was replaced and treatments were added in serial dilution from 256 to 32 µg/mL. Plates were subsequently incubated for 24 more hours and processed according to the manufacturer’s protocol for chemical induced cytotoxicity.

### Restoration assays

The effect of iron supplementation on PGG activity was determined for *A. baumannii* AB5075 by modifying Lin et al.'s iron restoration assays on *S. aureus*^14^. For the first half of the assay, PGG was mixed with solutions of cation-adjusted Mueller–Hinton Agar (CAMHA) and TSA at concentrations of 0.5 × MIC, 1 × MIC, and 2 × MIC (128, 256, and 512 µg/mL, respectively). A control condition with no PGG treatment was included. Each condition was tested in triplicate, along with media blank wells. Overnight culture of *A. baumannii* AB5075 was standardized to 5 × 10^5^ CFU/mL in CAMHB media at OD_600_. AB5075 working culture was spread onto each treatment plate, statically incubated at 35 °C for 24 h, and observed for presence of bacterial growth. At the 24-h mark, 1 mM of iron (II) sulfate solution or 1 mM of iron (III) sulfate solution was spread onto the PGG-treated wells. The plates were visually observed for presence of bacterial growth at 24 and 48-h timepoints.

### Development of resistance

Spontaneous development of resistance was tested using a method adapted from Ling et al.^53^. *A. baumannii* AB5075 culture was spread on TSA plates containing 0, 128, 256, and 512 µg/mL PGG and incubated for 24 h, after which colonies on each plate were counted.

Resistance over the course of serial passaging was tested using a method adapted from Maisuria et al.^54^. Briefly, a series of 21 microbroth dilution experiments for growth inhibition was carried with PGG or tetracycline against *A. baumannii* AB5075, using bacterial culture from the sub-MIC (0.5MIC) wells of each gradient to make the experimental culture for each subsequent experiment.

### Disclaimer

Material has been reviewed by the Walter Reed Army Institute of Research (WRAIR). There is no objection to its presentation and/or publication. The opinions and assertions contained herein are the private ones of the authors and are not to be construed as official or reflecting the views of the Department of Defense, the Uniformed Services University of the Health Sciences, the Department of the Army, or any other agency of the U.S. Government.

## Supplementary information

Supplementary file1

## Data Availability

The authors confirm that the data supporting the findings of this study are available within the article and its supplementary materials.
