# Supplementary material for: Pentagalloyl glucose from Schinus terebinthifolia inhibits growth of carbapenem-resistant Acinetobacter baumannii
Source: Sci Rep. 2020 Sep 18;10:15340. doi: 10.1038/s41598-020-72331-w (PMC7501240; doi:10.1038/s41598-020-72331-w)
Supplement: Supplementary file 1 — Supplementary file1 [file 41598_2020_72331_MOESM1_ESM.docx]

**Pentagalloylglucose from *Schinus terebinthifolia* inhibits growth of carbapenem-resistant *Acinetobacter baumannii***

Micah Dettweiler, Lewis Marquez, Michelle Lin, Anne M. Sweeney-Jones, Bhuwan Khatri Chhetri, Daniel V. Zurawski, Julia Kubanek, Cassandra L. Quave

**Supplementary Information**

Contents

[**Supplementary Figure S1.** Growth inhibition of CR *A. baumannii* AR-Bank #0035 by 1496 extracts from the QNPL chemical library (plotted by extract identification number ranging from 1-1881) at 256 µg/mL. 3](#_Toc48374990)

[**Supplementary Figure S2**. HPLC-FTMS ESI-negative chromatograms for (A) pentagalloyl glucose standard, (B) 429C-F8-PF11-SF4. 4](#_Toc48374991)

[**Supplementary Table S1**. Comparison of ^1^H NMR data 5](#_Toc48374992)

[**Supplementary Figure S3**. ^1^H NMR spectrum acquired in CD_3_OD (600 MHz). 7](#_Toc48374993)

[**Supplementary Figure S4.** ^1^H NMR spectrum acquired in DMSO-d_6_ (800 MHz). 8](#_Toc48374994)

[**Supplementary Figure S5.** ^13^C NMR spectrum acquired in DMSO-d_6_ (800 MHz). 9](#_Toc48374995)

[**Supplementary Figure S6.** COSY NMR spectrum acquired in DMSO-d_6_ (800 MHz). 10](#_Toc48374996)

[**Supplementary Figure S7.** HSQC NMR spectrum acquired in DMSO-d_6_ (800 MHz). 11](#_Toc48374997)

[**Supplementary Figure S8.** HMBC NMR spectrum acquired in DMSO-d_6_ (800 MHz). 12](#_Toc48374998)

[**Supplementary Figure S9.** Stained *A. baumannii* biofilm in pentagalloyl glucose treatment wells in A) biofilm formation inhibition experiment (64 µg/mL PGG) and B) biofilm eradication experiment (256 µg/mL PGG). C) shows staining of the media blank and D) shows staining of the vehicle control (DMSO). 13](#_Toc48374999)

[**Supplementary Figure S10.** PGG was evaluated for potential activity in the inhibition of **A**) biofilm formation and **B**) eradication of established biofilm. No significant difference was noted between PGG and the vehicle control (DMSO) at any of the test concentrations (0.5-64 µg/mL for biofilm inhibition; 2-256 µg/mL for eradication experiments). 14](#_Toc48375000)

[**Supplementary Figure S11.** Growth inhibition of *A. baumannii* AB5075 by PGG in TSA and CAMHA before and after iron (II) and iron (III) supplementation. 15](#_Toc48375001)

[**Supplementary Figure S12.** Optical density readings of PGG in 1 mM iron (II) and iron (III) sulfate supplemented media at 600 nm. 16](#_Toc48375002)

[**References** 17](#_Toc48375003)

**
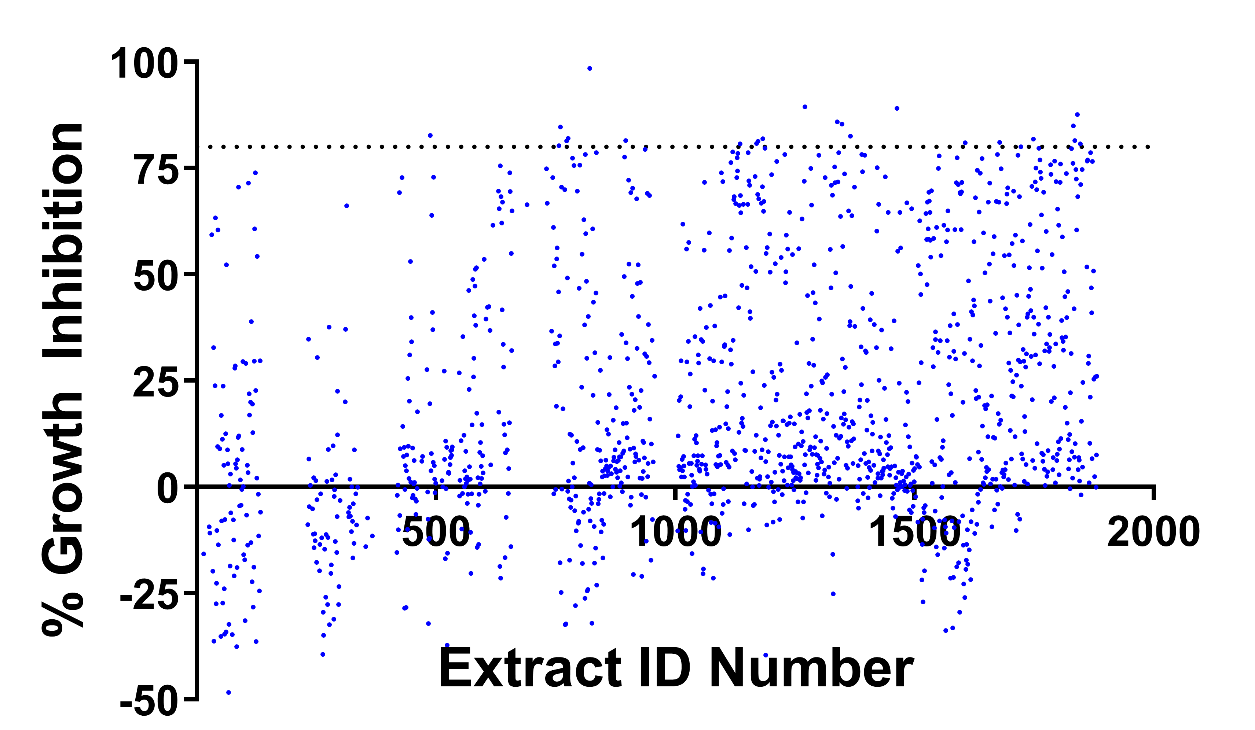
**

# **Supplementary Figure S1.** Growth inhibition of CR *A. baumannii* AR-Bank #0035 by 1496 extracts from the QNPL chemical library (plotted by extract identification number ranging from 1-1881) at 256 µg/mL.


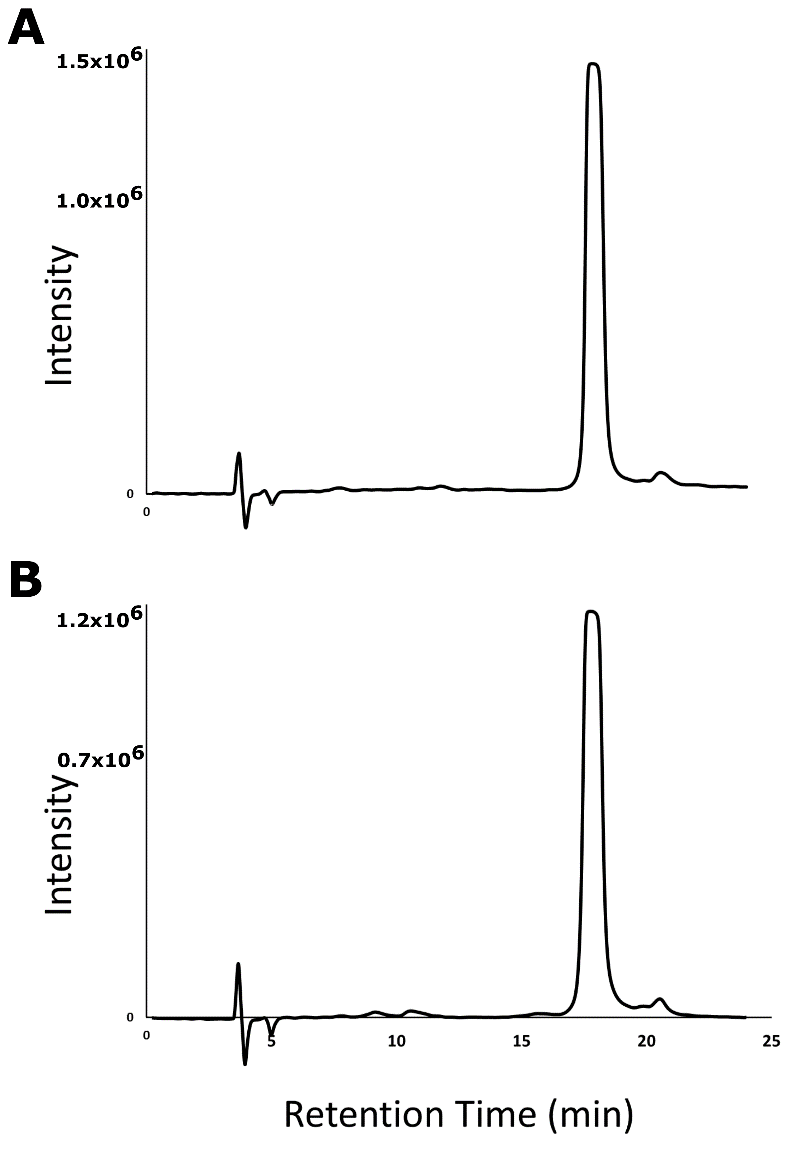


# **Supplementary Figure S2**. HPLC-FTMS ESI-negative chromatograms for (A) pentagalloyl glucose standard, (B) 429C-F8-PF11-SF4.

# **Supplementary Table S1**. Comparison of ^1^H NMR data

| **Position** | **δ_H_ in CD_3_OD, 600 MHz (experimental)** | **δ_H_ in CD_3_OD, 600 MHz (literature)** (1) | **δ_H_ in DMSO-d_6_, 800 MHz (experimental)** | **δ_H_ in DMSO-d_6_, 600 MHz (literature)** (2) |
| --- | --- | --- | --- | --- |
| **Glucose moiety** | | | | |
| 1 | 6.23, d (8.2) | 6.23, d (8.3) | 6.37, d (8.2) | 6.36, d (9.5) |
| 2 | 5.59, m (overlapped) | 5.58, dd (9.6, 8.3) | 5.42, m (overlapped) | 5.45, t (9.5) |
| 3 | 5.90, t (9.7) | 5.89, t (9.6) | 5.95, t (9.6) | 5.96, t (9.5) |
| 4 | 5.62, m (overlapped) | 5.61, t (9.6) | 5.44, m (overlapped) | 5.94, t (9.5) |
| 5 | 4.39 m (overlapped) | 4.40, m | 4.58, d (9.9) | 4.57, m |
| 6a | 4.51, d (12.5) | 4.51, d (12.2) | 4.30, m | 4.59, m |
| 6b | 4.39 m (overlapped) | 4.37, dd (12.2, 4.2) | 4.30, m | 4.30, m |
| **Galloyl moieties** | | | | |
|  | 6.90, 6.95, 6.98, 7.05, 7.11, s (each 2H) | 6.89, 6.94, 6.97, 7.04, 7.10, s (each 2H) | 6.77, 6.82, 6.85, 6.91, 6.97, s (each 2H) | 6.90, s, 10H |

**Supplementary Table S2**. Comparison of ^13^C NMR data

| **Position** | **δ_C_ in DMSO-d_6_, 800 MHz (experimental)** | **δ_C_ in DMSO-d_6_, 600 MHz (literature)** (2) |
| --- | --- | --- |
| -C=O | 164.0-165.5 | 164.4-165.9 |
| galloyl: C-3′, C-5′ | 145.4-145.7 | 145.8-146.1 |
| galloyl: C-4′ | 138.8-139.8 | 139.2-140.1 |
| galloyl: C-1′ | 117.3-119.0 | 117.9-119.4 |
| galloyl: C-2′, C-6′ | 108.7-109.0 | 109.2-109.5 |
| glucose: C-1 | 91.7 | 92.2 |
| glucose: C-2 to C-5 | 67.8-72.1 | 68.2-72.6 |
| glucose: C-6 | 61.5 | 61.9 |

# **Supplementary Figure S3**. ^1^H NMR spectrum acquired in CD_3_OD (600 MHz).

# **Supplementary Figure S4.** ^1^H NMR spectrum acquired in DMSO-d_6_ (800 MHz).

# **Supplementary Figure S5.** ^13^C NMR spectrum acquired in DMSO-d_6_ (800 MHz).

# **Supplementary Figure S6.** COSY NMR spectrum acquired in DMSO-d_6_ (800 MHz).

# **Supplementary Figure S7.** HSQC NMR spectrum acquired in DMSO-d_6_ (800 MHz).

# **Supplementary Figure S8.** HMBC NMR spectrum acquired in DMSO-d_6_ (800 MHz).

**
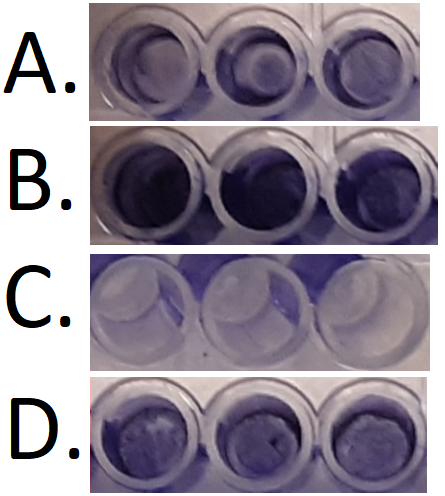
**

# **Supplementary Figure S9.** Stained *A. baumannii* biofilm in pentagalloyl glucose treatment wells in A) biofilm formation inhibition experiment (64 µg/mL PGG) and B) biofilm eradication experiment (256 µg/mL PGG). C) shows staining of the media blank and D) shows staining of the vehicle control (DMSO).


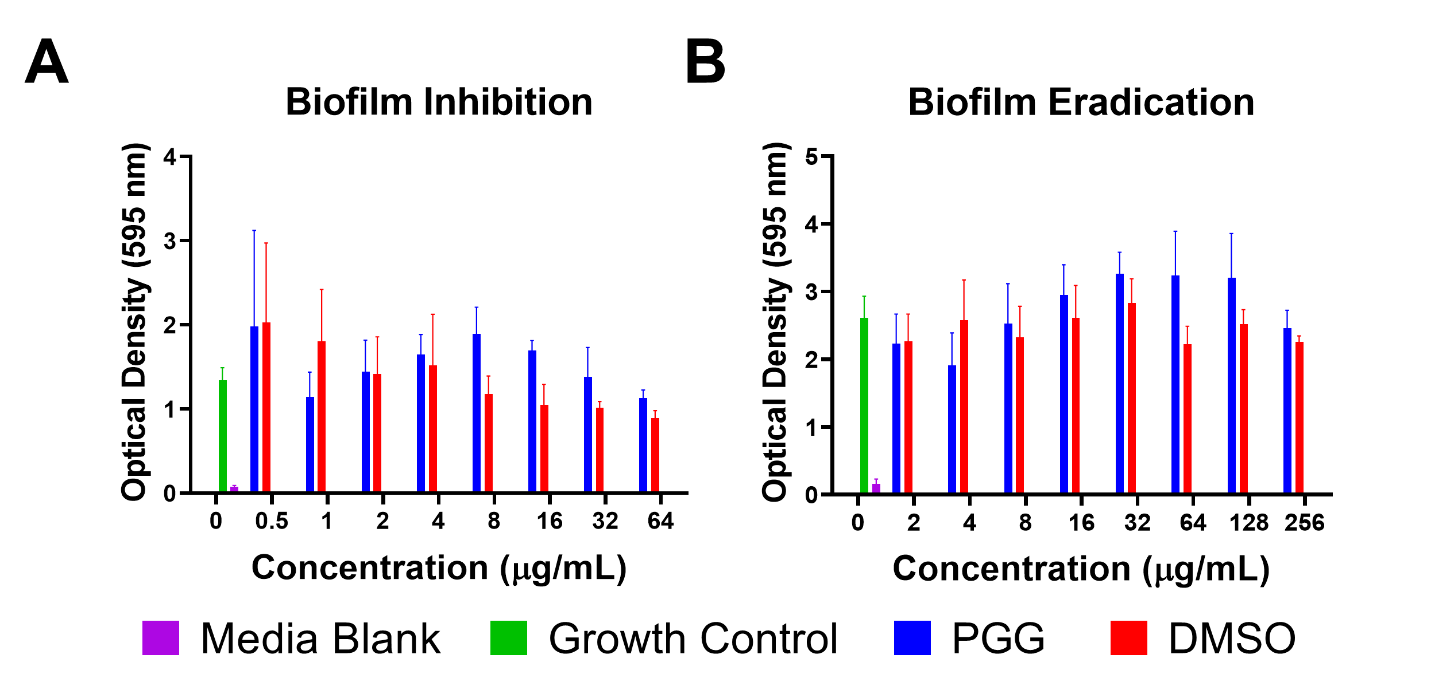


# **Supplementary Figure S10.** PGG was evaluated for potential activity in the inhibition of **A**) biofilm formation and **B**) eradication of established biofilm. No significant difference was noted between PGG and the vehicle control (DMSO) at any of the test concentrations (0.5-64 µg/mL for biofilm inhibition; 2-256 µg/mL for eradication experiments).


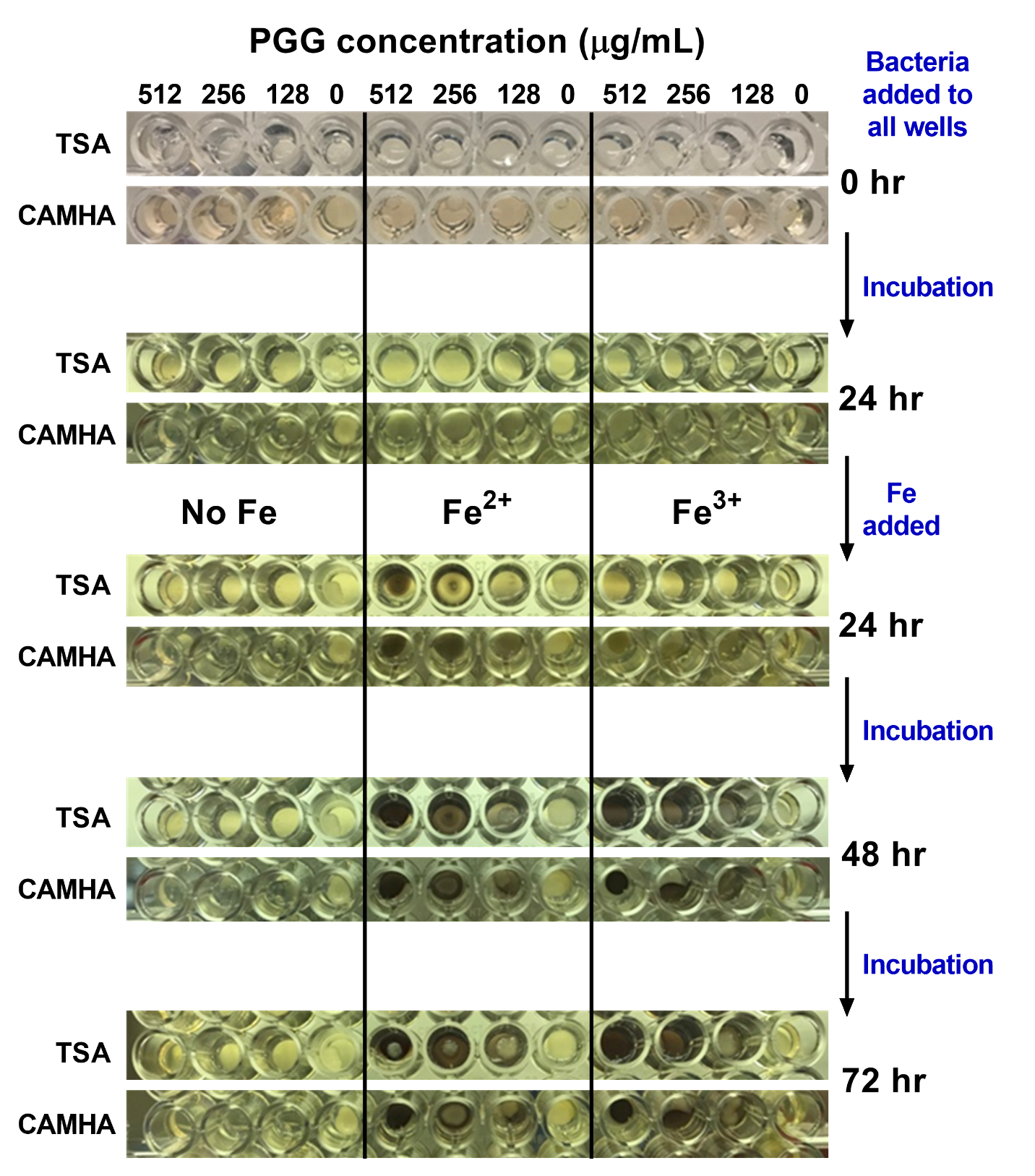


# **Supplementary Figure S11.** Growth inhibition of *A. baumannii* AB5075 by PGG in TSA and CAMHA before and after iron (II) and iron (III) supplementation.

**
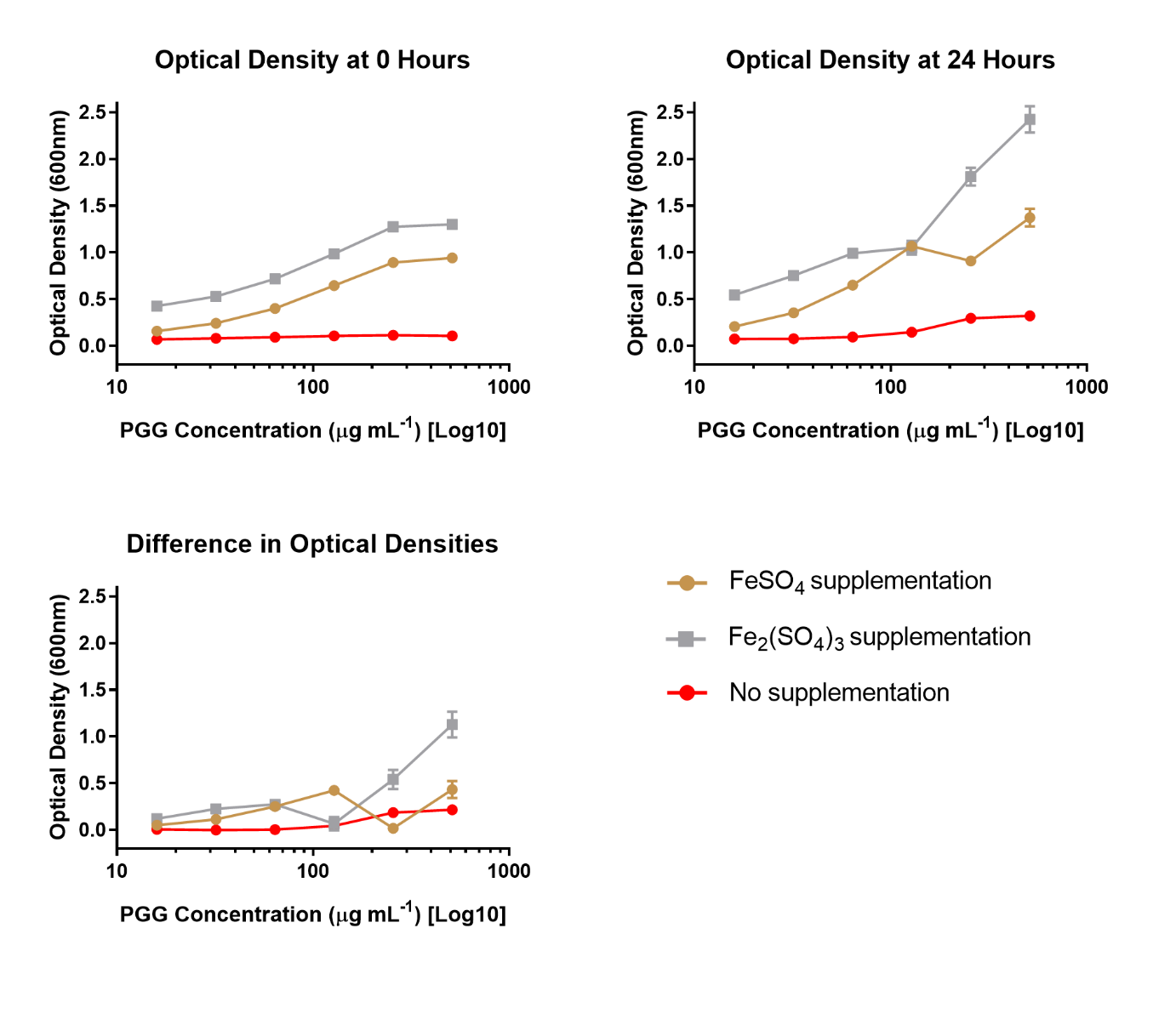
**

# **Supplementary Figure S12.** Optical density readings of PGG in 1 mM iron (II) and iron (III) sulfate supplemented media at 600 nm.

# **References**

1. Kant R, Yen CH, Lu CK, Lin YC, Li JH, Chen YMA. 2016. Identification of 1,2,3,4,6-Penta-O-galloyl-beta-D-glucopyranoside as a Glycine N-Methyltransferase Enhancer by High-Throughput Screening of Natural Products Inhibits Hepatocellular Carcinoma. International Journal of Molecular Sciences 17.

2. Zhao WH, Gao CC, Ma XF, Bai XY, Zhang YX. 2007. The isolation of 1,2,3,4,6-penta-O-galloyl-beta-D-glucose from Acer truncatum Bunge by high-speed counter-current chromatography. Journal of Chromatography B-Analytical Technologies in the Biomedical and Life Sciences 850:523-527.
